# Association of menstruation with life activities of adolescents in Abha, Saudi Arabia

**DOI:** 10.1097/MD.0000000000048708

**Published:** 2026-05-08

**Authors:** Shamsun Nahar Khalil, Aesha Siddiqui, Safar Abadi Alsaleem, Syed Esam Mahmood, Meaad Mohammed Abdullah Althabet, Awad Alsamghan, Ausaf Ahmad

**Affiliations:** a Department of Medical Studies, Faculty of Medical Studies (FMS), Bangladesh University of Professionals, Bangladesh; b School of Health, Victoria University of Wellington, New Zealand; c Department of Family and Community Medicine, College of Medicine, King Khalid University, Abha, Saudi Arabia; d Department of Family and Community Medicine, College of Medicine, King Khalid University, Abha, Saudi Arabia; e King Fahad Medical City, Riyadh, Saudi Arabia; f Department of Community Medicine, Kalyan Singh Government Medical College Bulandshahr, Bulandshahr, Uttar Pradesh, India.

**Keywords:** adolescents, dysmenorrhea, Saudi Arabia, school absenteeism

## Abstract

Menstrual disorders especially dysmenorrhea is a common problem in adolescent girls. Therefore, this study aimed to determine the prevalence of dysmenorrhea, and assess the association between dysmenorrhea and daily life interference among secondary-school girls in Abha, Saudi Arabia. This cross-sectional study was carried out in Abha city among 445 secondary-schoolgirls. A self-administered tool based on menstrual disorder of teenagers (MDOT) questionnaire was used for data collection. Descriptive statistics were computed for discrete variables, and mean and standard deviation were computed for continuous variables. Pearson’s chi-square test was used to test the association between variables of interest. Multivariable logistic regression analysis was performed to identify the menstrual symptoms that were independently associated with high interference in daily life activities and to identify the most affected daily life activity. *P*-values ≤ .05 was considered statistically significant to test the obtained results. One in 3 girls reported irregular menstruation. Dysmenorrhea was present in 75.3% of girls (45.1% reported mild, 29.9% reported moderate, and 25.0% reported severe pain). High interference in daily life activity due to dysmenorrhea was reported by 83.9% and around 70.3% missed school. In bivariate analysis, dysmenorrhea was significantly associated with limited physical activity (odds ratio = 1.67, 95% confidence interval 1.04–2.68). After adjusting for potential confounders, menstrual symptoms were independently associated with high interference in attending school (adjusted odds ratio = 2.61, 95% confidence interval 1.47–4.59). This study suggests that dysmenorrhea is highly prevalent among adolescent girls in Abha and is significantly associated with interference in daily life activities, particularly school attendance.

## 1. Introduction

Adolescence is the period of developmental transition between childhood and adulthood, involving multiple physical, intellectual, personality, and social developmental changes.^[1]^ One of the major physiological changes that take place in adolescent girls is the onset of menstruation. In adolescents, menstrual disorders are a common problem. The most common menstrual disorders reported from studies are irregular frequency of menstruation, premenstrual syndrome, irregular duration of menstruation, dysmenorrhea, polymenorrhoea, and oligomenorrhoea.^[2,3]^ Of these, dysmenorrhea is the commonest problem experienced by most adolescent girls. The prevalence of dysmenorrhea reaches up to 90% in some studies.^[4,5]^

Dysmenorrhea is defined as a sharp painful menstrual cramp in the lower abdomen and menorrhagia is heavy and prolonged menstrual bleeding. It is often accompanied by other symptoms including dizziness, fatigue, sweating, backache, headache, nausea, vomiting, and diarrhea all occurring just before or during the menstruation. The severity of menstrual pain ranges from moderate to severe among adolescents. Adolescent girls suffering from moderate to severe pain that is associated with several menstrual symptoms should be appropriately managed to reduce menstrual morbidity.^[5,6]^

Given the availability of effective medications, and despite the negative impact of dysmenorrhea, only 14% to 18% of adolescents seek medical advice, and about half take medications to alleviate their symptoms. Due to its importance, different treatments including pharmacological and nonpharmacological treatment approaches such as taking nonsteroidal anti-inflammatory drugs (NSAIDs), herbal, dietary therapies, yoga, meditation, and acupuncture have been used to lessen the effects of dysmenorrhea.^[6,7]^

Menstrual problems pose many physical, sociocultural, and economic challenges that may hinder their life activities mainly the ability to attend school or to participate in physical activities. Dysmenorrhea is a cause of frequent short-term work and school absenteeism and limitations on social, academic, and sports activities in adolescent girls.^[8–10]^ Girls who regularly miss school in menstruation, lose approximately 10% to 20% of the academic year.^[11,12]^ Loss of school time may affect their school performance as stated in an Ethiopian study where about 90% of girls stated that their academic performance declined after menarche.^[13]^

Although menstrual problems are common problems of adolescent females, they have attracted little or no attention in the public health agenda of most countries.^[14]^ Many factors are responsible for the low public attention accorded to menstrual health and its effects on the daily life of adolescents. These included cultural, social, and economic factors. People perceive the issue of menstruation as a personal affair that need not be discussed publicly. In some cultures, it is considered an impurity, and females are banished during their monthly cycle from household activities.^[15,16]^

Although studies have examined menstrual pain and symptoms in adult Saudi women, very few attempts have been made at exploring menstrual cycle problems among adolescents. While several studies have examined menstrual health in major Saudi cities like Riyadh and Jeddah^[17,18]^ data remains scarce regarding the Aseer region. This region is unique in its geography and conservative cultural setting, which may influence lifestyle factors and health-seeking behaviors differently than in the cosmopolitan centers. Furthermore, whereas the majority of local research has relied on the Moos Menstrual Distress Questionnaire or simple Visual Analogue Scales, this study utilizes the menstrual disorder of teenagers (MDOT) tool. This methodological distinction allows for a more specific quantification of how menstrual symptoms interfere with targeted daily activities, particularly school attendance and sports participation. Therefore, this study aimed to determine the prevalence of dysmenorrhea, and assess the association between dysmenorrhea and daily life interference among secondary-school girls in Abha, Saudi Arabia.

## 2. Methods

### 2.1. Participants, setting, and design

**Figure 1.**
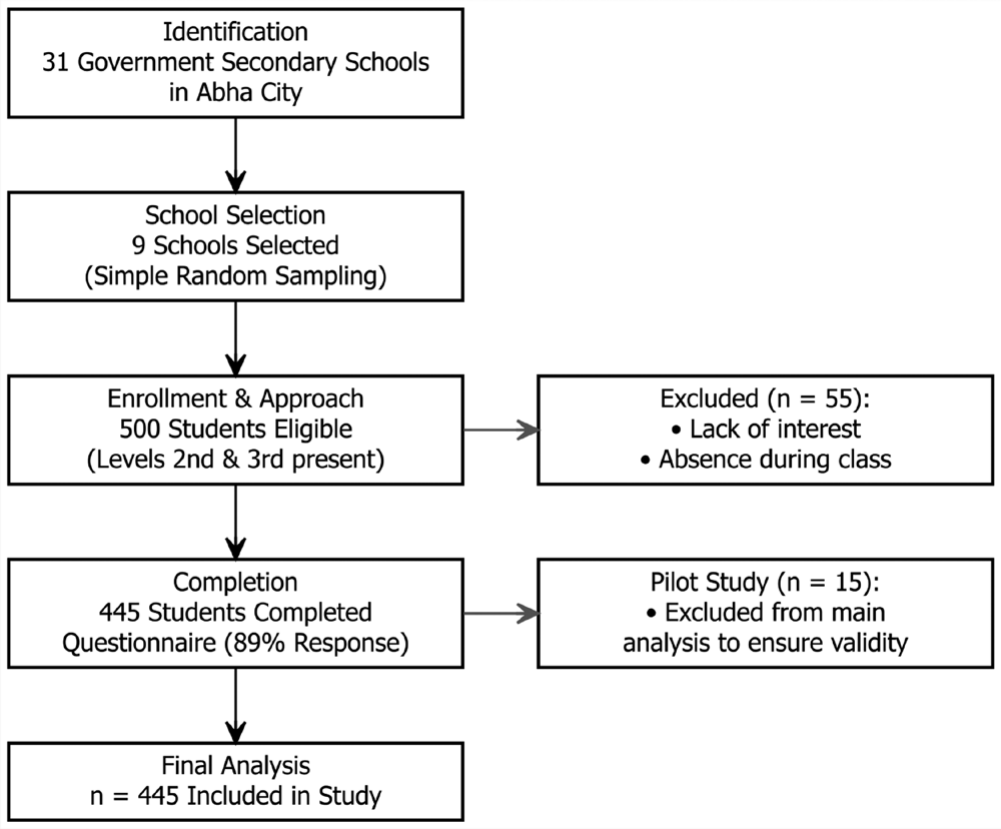
Flowchart representation of the participant flow.

This cross-sectional study was carried out in Abha City, Asir province of Saudi Arabia. The target population was female secondary-school students during the academic year 2018 to 2019.Using the Roasoft o445nline sample size calculator, the sample size was calculate on assumption that the total population of female secondary school students is 5420, the lowest prevalence of menstrual disorders is 19.3%,^[2]^ 95% confidence interval and 5% acceptable errors, the calculated sample size was 230. The city has a total of 31 female government secondary schools. A multistage cluster sampling technique was used to select the schools. Nine schools were selected for the study using simple random sampling. To ensure robust results and account for the design effect of cluster sampling, all students in the selected classes were invited to participate, resulting in a pool larger than the minimum calculation. All individual students in levels second and third (age 14–19 years) who were present in the school on the day of data collection were included invited to participate. Of the 500 students eligible and approached in these schools, 445 completed the questionnaire, yielding a response rate of 89%. Reasons for nonparticipation were primarily lack of interest or absence during the specific class period. No systematic exclusion of specific groups was applied. The data was collected between March to May 2019. A visual representation of the participant flow, including eligibility, approach, completion, and reasons for nonparticipation, is detailed in Figure 1.

### 2.2. Research tool

The MDOT designed by^[5]^ was used as the basis to develop a research tool in Arabic language by an authorized translator. To adapt the questionnaire to Saudi cultural preferences, 2 questions related to sexual behavior and one question on casual paid work during periods were deleted. These were excluded to ensure cultural appropriateness, as the study population consists of unmarried adolescent girls in a conservative society where sexual activity is culturally proscribed and unlikely to be reported. Consequently, these questions were deemed irrelevant to the menstrual symptomatology of this specific cohort. Pretesting was done on fifteen students to ensure comprehensibility, linguistic accuracy, and time taken for completion. The validity and reliability of the questionnaire was established by calculating the crohnbach alpha, which was calculated as 0.78. The data from the pilot study was not included in the main study.

Data was gathered on the student’s age, age at menarche, regularity of the cycle, length, and duration of the cycle, amount of bleeding, pain during menstruation, the severity of the pain, associated physical and emotional symptoms experienced during the menstrual cycle, and medication used, if any. Questions were included to measure the interference with menstrual symptoms and dysmenorrhea in daily activities. The menstrual-related experiences of the participants over the past 6 months were collected. For the study, regular menstruation is defined as a cycle repeated once every 28 to 32 days with a duration of 5 to 7 days.^[5]^ Dysmenorrhea is defined as crampy pelvic pain, abdominal pain, or backache on the first day of the menstrual period and lasting up to 3 days.^[19,20]^ Heavy bleeding is defined as having to use 2 pads at a time.^[20]^

To measure the intensity of menstrual pain, a scale of 0 to 10 was used. Zero represents no pain at all and 10 indicates severe pain. The students were asked to rate the intensity of pain by marking the number. The score marked on the scale was classified into mild dysmenorrhea if it was between 1 and 3, moderate between 4 and 7, and severe between 8 and 10.^[5]^

Self–perceived interference in 6 daily life activities was measured. These activities included school attendance, completing school work, relationships with family and friends, social activities, sports & exercise. A rating scale of 0 to 10 was used. A score of 5 to 10 was classified as high interference and 0 to 4 as low interfoterence.^[5]^

### 2.3. Method of data collection

Data were collected through a self-administered questionnaire. A letter was issued to the selected school heads regarding all information about the study. Upon the school head’s agreement to participate in the study, data collection was conducted by the researchers 3 days per week between March to May 2019. A teacher was present to assist in the activities during data collection. The students were informed of the objectives of the study and how to fill in the questionnaire. They were explained about dysmenorrhea, its associated symptoms, how to rate the intensity of pain, and the rating method of menstrual interference on their daily life activities. Before obtaining verbal consent, all students were informed that participation in the study was voluntary, the questionnaire would be anonymous, and the data collected would be strictly confidential and used only for this study. The ethical permission was obtained from the research ethical committee at King Khalid University (ECM 2020-1204).

### 2.4. Analysis of data

The collected data were verified by hand before computerized data entry. Descriptive statistics, such as frequencies and percentages, are provided for various characteristics of the respondents, including age, nationality, school grade, parents’ education, family income, menarche, menstrual pattern, symptoms, and medication use. Further Descriptive statistics are provided for the degree of interference of menstrual symptoms in daily life activities, categorized as low interference and high interference. Chi-square tests are employed to examine the relationship between dysmenorrhea and various life activities, such as school attendance, sports and exercise, completing school work, relations with family and friends, and social activity. Odds ratios (OR) with 95% confidence intervals (CI) are also calculated to measure the strength of association between dysmenorrhea and each life activity.

Chi-square tests are also used to explore the relationship between the severity of pain and interference in life activities.

Missing data was minimal (<5% for core variables). We utilized mean imputation for continuous variables with missing values to maintain sample size. A multivariable logistic regression model was constructed to assess the association between menstrual symptoms and high interference in daily activities. The model was adjusted for potential confounders including age, age at menarche, and family income. The Statistical Package for Social Sciences (SPSS) software version 22.0 was used for data analysis.

## 3. Results

A total of 445 adolescent girls were included in the study. Their mean age was16.27 ± 1.45 years, with a minimum age of 14 and a maximum of 19 years.

Table 1 describes the basic and menstrual characteristics of the study group. Girls ≤ 15 years comprised 30.8%,16 to 17 years were 46.1% and ≥ 18 years were 23.15% of the study population. Most of the girls (83.8%) were Saudi. Regarding the parental education level,19.8%mothers and 35.7% fathers had a university education. More than half of the students reported a family income of between 5000 and 10,000 SR per month. The mean age at menarche was 12.9 ± 1.28, with a minimum of 9 years and a maximum of 16 years. One in 10 girls reported age at menarche <12 years and almost two-thirds had menarche between 12 and 13 years, and about 1 in 4 had menarche at age more than 13 years.

**Table 1:** Sociodemographic and menstrual characteristics of study participants (n = 445).

Characteristics	Number (%)
Age of the respondents	
≤15 yr	137 (30.8)
16–17	205 (46.1)
≥18 years	103 (23.1)
Nationality	
Saudi	373 (83.8)
Non Saudi	72 (16.2)
School grade	
Level 2	173 (38.9)
Level 3	272 (61.1)
Mother’s education	
High school	357 (80.2)
University	88 (19.8)
Father education	
High school	286 (64.3)
University	159 (35.7)
Income of the family	
<5000 SR	115 (25.8)
5000–10,000	240 (53.9)
>10,000SR	90 (20.3)
Menarche	
<12	45 (10.1)
12–13	279 (62.7)
>13	121 (27.2)
Menstrual pattern	
Regular	294 (66.1)
Irregular	151 (33.9)
Cycle length	
21–28 d	210 (71.4)
>28 d	84 (29.6)
Heavy bleeding	
No	237 (53.3)
Yes	208 (46.7)
Dysmenorrhea	
Present	335 (75.3)
Absent	110 (24.7)
Severity of pain (n = 335)	
Mild pain (1–3)	151 (45.1)
Moderate pain	100 (29.9)
Severe pain	84 (25.0)
Medication use	
Yes	274 (61.6)
No	171 (38.4)
Symptoms	
Change in appetite	236 (53.0)
Nausea	174 (40.9)
Bloating	189 (42.5)
Diarrhoea	175 (39.3)
Vomiting	128 (28.8)
Indigestation and heartburn	113 (25.4)
Pelvic pain	233 (52.4)
Leg pain	167 (37.5)
Low back pain	322 (72.4)
Headache	246 (63.3)
Dysuria	185 (41.6)
Itching	161 (36.2)
Dizziness	157 (33.3)
Low mood	250 (56.2)

One in 3 girls reported irregular menstruation. Among those with a regular pattern of menstruation (66.1%), a cycle length of 21 to 28 days was reported by 71.4% and more than 28 days by the remaining 29.6%. Heavy bleeding was reported by 46/7% of the girls. Dysmenorrhea was present in 75.3% of girls. The severity of pain among those who reported dysmenorrhea varied. from mild (45.1%), moderate (29.9%), and severe (25.0%) dysmenorrhea. Among those with dysmenorrhea, the most common medication used was NSAIDs (50%), while 30% used herbal preparations.

The prevalence of associated symptoms is detailed in Table 1 (calculated based on all 445 participants). The most frequently reported gastrointestinal symptoms were poor appetite (53%) and bloating (42.5%), while musculoskeletal pain, specifically low back pain (72.4%), was the most common somatic complaint. Other symptoms reported were low mood by 56.2%, dizziness (33.3%) and itching by 36.2%.

Table 2 shows the interference of menstrual symptoms in the daily life of the respondents. The symptoms which highly interfere in life were mood disturbance (80%), dysmenorrhea (83.9%), fatigue(75.5%), feeling unwell (73.7%), and heavy blood flow(51.9%). This interference in daily life occurred for 17% of girls in all or most periods(33%), while 44.7% reported the interference in some periods.

**Table 2:** Prevalence and degree of interference of menstrual symptoms in daily life activities among study participants (n = 445).

Variables	Degree of interference in daily life	
	Low interference Number (%)	High Interference Number (%)
Menstrual symptoms		
Mood disturbance	88 (19.8)	357 (80.0)
Dysmenorrhea	54 (16.1)	281 (83.9)
Tiredness/Fatigue	109 (24.5)	336 (75.5)
Feeling unwell	117 (26.3)	328 (73.7)
Heavy blood flow	214 (48.1)	231 (51.9)

The association between of dysmenorrhea on life activities is presented in Table 3. A total of 313 (70.3%) miss school. On average, all girls, irrespective of whether they suffer from menstrual pain or not, miss 1 day of school. However, the mean number of missed school days for the girls with dysmenorrhea is 2 days. Seventy-seven (17.3%) girls miss school in all periods, 149 (33.5%) in most periods, and 20 girls(4.5%) reported never missing school. Statistically significant differences were observed between girls with and without dysmenorrhea in 2 daily life activities, namely attending school and sports/physical activities. In girls reporting dysmenorrhea, there was a disturbance in school attendance among 86.5% as compared to 13.5% in those without dysmenorrhea (*P* < .001) (OR = 2.95, 95% CI 1.78–4.91). In the group with dysmenorrhea, there was a disturbance in doing physical activity among 81.1% as compared to 18.9% in those without dysmenorrhea (*P* = .03, OR = 1.67, 95% CI 1.04–2.68). The other daily life activities of completing school work, relationships with family and friends, and social activities did not show a significant difference between the 2 groups however in all activities, a higher proportion of girls in the group with dysmenorrhea reported interference as compared to those without dysmenorrhea.

**Table 3:** Association between dysmenorrhea and daily life activities among study participants (n = 445).

	Dysmenorrhoea Number (%)		Chi-square, *P* value	Crude OR & 95% CI
Life activities	No	Yes		
School attendance				
Low interference	87 (31.6)	188 (68.4)	18.51, < .001	2.95 (1.78–4.91)
High interference	23 (13.5)	147 (86.5)		
Sports and exercise				
Low interference	80 (28.0)	206 (72.0)	4.55, .03	1.67 (1.04–2.68)
High interference	30 (18.9)	129 (81.1)		
Completing school work				
Low interference	82 (27.3)	218 (72.7)	3.38, .06	1.57 (0.96–2.55)
High interference	28 (19.3)	117 (80.7)		
Relation with family				
Low interference	74 (24.3)	231 (75.7)	0.11, .74	0.92 (0.58–1.46)
High interference	36 (25.7)	104 (74.3)		
Relation with friends				
Low interference	79 (24.6)	242 (75.4)	0.01, .93	0.97 (0.61–1.58)
High interference	31 (25.0)	93 (75.0)		
Social activity				
Low interference	77 (26.1)	218 (73.9)	0.89, .343	1.25 (0.78–1.99)
High interference	33 (22.0)	117 (78.0)		

CI = confidence interval, OR = odds ratio.

The relationship between the severity of pain and interference in life activities is presented in Table 4. Among all 6 activities, the degree of pain highly interferes with attending school, completing school work, and relations with friends. Multivariable logistic regression (adjusted for age, menarche age, and socioeconomic status) indicated that dysmenorrhea emerged as the strongest predictor, as participants reporting dysmenorrhea had 2.62 times higher odds of experiencing high interference compared to those without it (aOR = 2.62; 95% CI: 1.49–4.62; *P* = .001). Furthermore, heavy bleeding (aOR = 2.31; 95% CI: 1.37–3.91; *P* = .002) and a general feeling of being unwell (aOR = 2.00; 95% CI: 1.10–3.72; *P* = .028) were also found to be significant independent predictors, doubling the odds of high life interference. Consequently, among the specific daily activities evaluated, the analysis revealed a highly significant independent association between these menstrual symptoms and interference with attending school (aOR = 2.61; 95% CI: 1.47–4.59; *P* = .001) (Table 5).

**Table 4:** Relationship between menstrual pain severity and degree of interference in daily life activities among study participants (n = 445).

Life activity		Severity of pain			
	No pain	Mild	Moderate	Severe	Chi-square *P*-value
Attending school					
Low interference	87 (31.6)	100 (36.4)	54 (19.6)	34 (12.4)	33.93, <.001
High interference	23 (13.5)	51 (30.0)	46 (27.1)	50 (29.4)	
Sports and exercise					
Low interference	80 (28.0)	94 (32.9)	63 (22.0)	49 (17.1)	5.05, .16
High interference	30 (18.9)	57 (35.8)	37 (23.3)	35 (22.0)	
Completing school work					
Low interference	82 (27.3)	109 (36.3)	63 (21.0)	46 (15.3)	11.12, .01
High interference	28 (19.3)	42 (29.0)	37 (25.5)	28 (26.2)	
Relation with family					
Low interference	74 (24.3)	111 (36.4)	69 (22.6)	51 (16.7)	4.21, .24
High interference	36 (25.7)	40 (28.6)	31 (22.1)	33 (23.6)	
Relation with friends					
Low interference	79 (24.6)	117 (36.4)	75 (23.4)	50 (15.6)	9.21, .027
High interference	31 (25.0)	34 (27.4)	25 (20.2)	34 (27.4)	
Social activity					
Low interference	77 (26.1)	108 (36.6)	60 (20.3)	50 (16.9)	6.02, .11
High interference	33 (22.0)	43 (28.7)	40 (26.7)	34 (22.7)	

**Table 5:** Multivariable logistic regression analysis of menstrual symptoms independently associated with high interference in daily life activities among study participants (n = 445).

Symptoms	*P*-value	aOR	CI
Dysmenorrhea	.001	2.62	1.49–4.62
Heavy bleeding	.002	2.31	1.37–3.91
Feeling unwell	.028	2.00	1.1–3.72
Daily activity			
Attending school	.001	2.61	1.47–4.59

aOR = adjusted odds ratio, CI = confidence interval.

## 4. Discussion

### 4.1. Sociodemographic and menstrual characteristics

This study has for the first time in the Aseer region, established the distinctive experience of menstruation and its interference with daily life activities for a sample of Saudi teenage girls. There are many important milestones in the life of a girl as she grows to become a woman. One of the most important events during sexual development is the first episode of menstrual blood flow described as menarche. This important developmental milestone in females has been found to vary greatly across countries.^[21]^ Globally, the average age of onset of menarche is 12.4 years.^[22]^ In the present study age at menarche was reported 12.9 years, which is similar to the global average and also to that reported from other countries like Nigeria,^[23]^ Hong Kong,^[24]^ Lebanon,^[25]^ and Saudi Arabia.^[26]^

The menstrual cycle is recognized as a vital sign that is indicative of the overall health of young females.^[22]^ The average menstrual cycle is 28 days, can range between 21 and 35 days, and lasts for a period of 3 to 7 days.^[5]^ Menstrual cycle variability or regularity is associated with a variety of factors.^[27]^ Similar to teenagers around the world, our study reported 1 in 3 girls with irregular menstruation or a cycle length of more than 28 days.^[5,28]^ However, heavy bleeding was reported by a higher proportion of girls as compared to other studies.^[28]^ This may be due to the subjective nature of this finding.

Menstruation may be associated with various symptoms occurring before or during menstrual flow. Commonly reported symptoms in our study included poor appetite, nausea, bloating, vomiting, indigestion, and heartburn. The pain was reported by the majority of the girls which characteristically was low back pain, leg pain, headache, and dysuria. Other symptoms included low mood, dizziness, and itching. The menstrual symptoms reported in our study are universal and comparable patterns of menstrual illness reported by other studies.^[1,20,29,30]^

Dysmenorrhea is a common problem among adolescent girls cutting across regional and social boundaries.^[1,5,13,20]^ As many as 90% of adolescent females worldwide report suffering from dysmenorrhea.^[10]^ However, our study reported that more than 75% of girls suffer from dysmenorrhea.

The high prevalence of dysmenorrhea (75.3%) observed in this study aligns with recent national findings, such as Jareebi et al (2025), who reported a prevalence of 87%.^[31]^

Previous Saudi researches have reported rates ranging from 60.9% to 92.3%.^[17,32–35]^

This high rate may be attributed to lifestyle factors prevalent among Saudi adolescents, including sedentary behavior and dietary habits, which have been linked to inflammatory markers and menstrual pain.

The influence of dysmenorrhea is largely dependent upon the severity of pain. The severity of menstrual pain is described as ranging from mild to severe.^[5]^ In the current study, 25 % of girls with dysmenorrhea described it as severe. This is higher than the figure of 10% to 20% in earlier studies.^[9,10]^ The disparity in the reported pain severity may be linked to individual variation in pain tolerance along with cultural differences in pain perception^[36]^

Among those with dysmenorrhea, the most common medications used were NSAIDs, followed by herbal preparations. Though the most commonly used method, NSAID use in this population was lower than that reported in an Australian study.^[5]^ The pattern of medication use, both herbal and conventional, is converse to that reported from other countries^[27,30]^ where more than sixty percent of the respondents use home remedies as a primary management option followed by NSAIDs. These differences may be due to the availability and accessibility of over the counter pain medications in Saudi Arabia.

1. Interference with daily life activities: Many menstrual symptoms interfere with the daily life of the adolescent. These include dysmenorrhea, mood disturbance, fatigue, feeling unwell, and heavy blood flow^[5]^ Many studies have reported that school absenteeism, poor concentration, low mood, physical inactivity, and disturbed behavior are the most important effects of dysmenorrhea.^[5,11,14,29,30]^
  i School absenteeism: In an Australian study, girls reported 26% school absence,^[5]^ while it was higher in Tanzania, Iraq, and Nigeria^[1,20,23]^ Similarly our study showed that the most profound association of dysmenorrhea was on school activities like attending school and completing school work. A total of 70.3% of the girls missed school due to dysmenorrhea. This school absenteeism is significantly related to pain intensity. A study showed that girls could miss up to 4 consecutive days of school every month because of their periods, meaning that they missed 10% to 20% of school time.^[12]^Similarly in Bangladesh, absentee girls missed an average of 2.8 days each menstrual cycle, constituting approximately 16% of the academic year.^[11]^ In our study girls missed an average 2 days of school during each menstrual period, which amounts to approximately 8.0% of the total academic time each year. Although this absenteeism is high, however it is less than other countries as cited above. High absenteeism may seriously impact the academic achievement of girls,^[37]^ however, this is not addressed in the current study and deserves further investigation.
  ii Association with physical activity: In a recent study from India, more than 3 fourth of the study participants stated that their menstrual symptoms were associated with poor physical activity.^[37]^ Another Indian study showed that more than half of students avoided participation in sports activities at school due to menstruation.^[38]^ Similar findings were observed by Khamdan et al^[39]^ who reported that 62% of girls stopped exercising during their period. Our findings are consistent with these studies. High interference of menstrual symptoms in daily activities may be attributed to the cultural taboos associated with menstruation. Another reason of low physical activity in our study group may be due to limited options of physical activity available to the girls, coupled with school absenteeism during the menstrual period. Many studies have pointed out that the severity of menstrual pain is the most prominent cause of interference in life activities. Severe pain was reported by 25 % of our respondents and 81% of those missing school attributed it to severely painful periods. Our findings are supported by other studies where the pain was described as severe and distressing enough to stop normal daily functions at work, home, or school by 10% to 20% of the respondents.^[9,10]^ Other studies have mentioned the nonavailability of menstrual hygiene facilities in schools like unlocked toilets, unavailability of sanitary pads in schools, feelings of shame, anxiety about leakage, and staining of their uniform as reasons for school absenteeism.^[11,29,33]^ In the current study, we did not explore these areas which are essential to foster a better understanding of the reasons for high school absenteeism among the study group. A further in-depth study using a mixed method would help to identify this.

## 5. Limitation

This study has several limitations. First, the cross-sectional design precludes causal inferences between dysmenorrhea and life interference. Second, data was self-reported, which introduces potential recall bias and social desirability bias, particularly regarding hygiene practices. Third, while we adjusted for basic confounders, unmeasured factors such as BMI, detailed nutritional status, and genetic history were not included in the regression analysis. This study was conducted in one city in the Kingdom of Saudi Arabia. As a result, the findings of the study cannot be generalized to all Saudi adolescent school girls. Additionally, restricting the study to only government schools introduces potential selection bias. Furthermore, defining heavy bleeding subjectively as ‘having to use two pads at a time’ represents a nonstandardized measurement limitation. Base on the important findings from this study, we are providing here some recommendations. Firstly, Health education on menstrual problems targeting female adolescents and their parents, and routine screening for menstrual problems by healthcare providers, can help prevent the loss of invaluable school time. Future research aimed at developing short screening tools that can be used by primary health carers and teenagers to identify girls requiring further investigation of menstrual disturbance. This will hopefully facilitate the earlier diagnosis of disorders that require management. Considering the role of physical activity, there should be better systems in place to encourage young girls to practice physical activity on regular basis and providing facilities for that at schools. Last but not the least awareness programmes and encouragement of school girls to talk to their teachers, physicians as well as mothers about their menstrual abnormalities to ensure them and improve their quality of life.

## 6. Conclusion

This study indicates a significant association between dysmenorrhea and reduced quality of life among adolescent girls in Abha. Dysmenorrhea was strongly correlated with school absenteeism and reduced participation in physical activities.

## Acknowledgments

All authors acknowledge with gratitude the support of the school authority for extending their cooperation during this study. We also thank all members of our team involved in data collection. Finally, we sincerely thank all the respondents who participated in this study.

## Author contributions

**Conceptualization:** Shamsun N. Khalil, Aesha Siddiqui.

**Data curation:** Shamsun N. Khalil, Aesha Siddiqui.

**Funding acquisition:** Syed Esam Mahmood.

**Formal analysis:** Shamsun N. Khalil, Aesha Siddiqui, Ausaf Ahmad.

**Investigation:** Shamsun N. Khalil, Aesha Siddiqui, Meaad Mohammed Abdullah Althabet, Awad Alsamghan.

**Methodology:** Shamsun N. Khalil, Aesha Siddiqui, Meaad Mohammed Abdullah Althabet, Ausaf Ahmad.

**Project administration:** Syed Esam Mahmood.

**Resources:** Shamsun N. Khalil, Meaad Mohammed Abdullah Althabet, Awad Alsamghan.

**Software:** Shamsun N. Khalil.

**Supervision:** Shamsun N. Khalil, Safar Abadi Alsaleem.

**Validation:** Shamsun N. Khalil, Aesha Siddiqui, Safar Abadi Alsaleem, Awad Alsamghan.

**Visualization:** Shamsun N. Khalil, Aesha Siddiqui, Safar Abadi Alsaleem.

**Writing – original draft:** Shamsun N. Khalil, Aesha Siddiqui.

**Writing – review & editing:** Safar Abadi Alsaleem, Syed Esam Mahmood, Ausaf Ahmad.
